# Practices of patients consuming antibiotics and knowledge about antibiotic resistance in Hail region – Saudi Arabia

**DOI:** 10.2144/fsoa-2019-0054

**Published:** 2019-09-27

**Authors:** Imaan Benmerzouga, Seham A Al-Zammay, Maha M Al-Shammari, Sitah A Alsaif, Taghreed M Alhaidan, Mohamad Aljofan

**Affiliations:** 1 Department of Clinical Pharmacy, College of Pharmacy, University of Hail, Hail, Kingdom of Saudi Arabia; 2 Department of Biomedical Sciences, West Virginia School of Osteopathic Medicine, Lewisburg, West Virginia, USA; 3 Department of Biomedicine, School of Medicine, Nazarbayev University, Nur-Sultan 010000, Kazakhstan

**Keywords:** antibiotic misuse, antibiotic resistance, attitude, knowledge, practice, survey

## Abstract

**Aim:**

Antibiotic misuse is considered one of the major causes of antimicrobial resistance.

**Objective:**

The study aims to investigate the practices of antibiotic misuse in the region of Hail, Saudi Arabia and the extent of people awareness of antibiotic resistance.

**Methods:**

Participants ≥18 years of age of both genders were recruit by convenience sampling from different public places.

**Results:**

Out of 500 participants, 26% obtained their antibiotic without a prescription, 27% took antibiotics for unknown infections and only 34% completed antibiotic course. A total of 36.2% did not know about antibiotic resistance and its rise, but the majority were willing to learn. The results warrant further study into malpractice with a wider geographic area and sample size to generalize the results for the whole country.

## Lay abstract

Antibiotics are a group of drugs that treat different bacterial infections. However, inappropriate use of antibiotics such as overuse (used when they are not needed) or use for the wrong reason can lead to antibiotic resistance (bacteria that are harder to treat). Patient knowledge of antibiotics is considered an important contributing factor for antibiotic misuse. Thus, this study was conducted to evaluate patient knowledge about antibiotic misuse and resistance among a population of Hail in the northern part of Saudi Arabia.

Antibiotics have been around since the 1950s, helping people survive what could be fatal infections and ultimately live longer. Many antibiotic classes were discovered during the ‘golden era’ of the 1950s to 1970s [1]. Antibiotics have multiple mechanisms of action, including inhibiting cell wall synthesis or DNA synthesis, among others [2]. This in turn leads to the death (cytotoxic) or stalling (cytostatic) actions of the drugs on the microorganisms. With their high success rate as pharmacological agents came their increased rate of ineffectiveness. This comes as no surprise, for example, we know that the incorrect use of penicillin will compromise its effectiveness [2].

Despite technological advancements and public awareness of such an epidemic, many practices remain resistant to change. For instance, in Europe, a study by Grigoryan *et al.* that surveyed ∼15,485 participants across 19 European countries reported a consistent association between prescribed use and self-medication [2]. The practice of self-medication stems from keeping the ‘leftover’ antibiotics instead of their disposal. On the other hand, in developing countries, antibiotics are readily available as over the counter medications [3]. In the USA, the number of antibiotics prescribed in some states exceeds the population of the state, with more than one prescription per person per year [3]. Inappropriate use and prescription of antibiotics have contributed to the promotion of antibiotic resistance (AR).

With AR on the rise, the United Nations has called for a global action plan to combat antimicrobial resistance. AR poses a major threat to human and animal health, crop production and development. The compounding effect of AR can be observed with the epidemic of newborn deaths (∼200,000 deaths/year) that are a result of infections that do not respond to current antibiotics, as well as the rise of the number of cases of untreatable bacterial infections [4–6]. Strains of bacteria that have evolved resistance to multiple classes of antibiotics have emerged and pose a serious health threat [7–9].

Many factors have been attributed to the rise in AR including overuse, misuse and the availability of few new antibiotics [8]. Overuse and misuse can be attributed to both practitioners and patients. Overuse or inappropriate prescribing has been documented in 30–50% of cases and a large number of antibiotics prescribed in the intensive care unit (ICU) are rendered unnecessary [3,10]. On the other hand, the misuse stems from the access and availability of antibiotics over the counter in some countries or their online purchase in countries where there is restricted access to antibiotics [11,12].

It is evident that AR is a multifactorial problem and coordinated efforts among various parties including patients is necessary to initiate headway in combating this deadly inevitable evolutionary phenomenon. Therefore, in this study, we aimed to investigate the practices of people who consume antibiotics and the extent of their awareness of AR in the region of Hail, Saudi Arabia. These data present one of the first reports of antibiotic use and AR awareness in the region of Hail, a small town in the northern region of Saudi Arabia.

## Methods

### Ethics approval

A submission including full project proposal was made to the Scientific Research Ethical Committee (SREC) at the University of Hail in the Kingdom of Saudi Arabia. The proposal was reviewed and unconditionally approved by the Committee. Approval number H-2016-046.

### Calculating sample size

To calculate the sample size of the study, the absolute error was estimated to be 5% and a 95% confidence level was used. Therefore, the sample size calculated for this study using the aforementioned information was 368 participants. In addition, the attrition rate (i.e., to consider nonrespondents) was 10%. Therefore, the required number of participants was 405.

### Research design & data collection

This is a cross-sectional questionnaire-based study that was carried out from October 2016 to February 2017. Participants were randomly selected by convenience sampling method at public places (technical schools, parks, malls, hospitals and the university) all over the city of Hail.

Participants consented to participate after they were given full details of the study and its intended aims. All participants were made aware that this study is for research purposes only and their participation was voluntary. They were not asked for their names or contact information, ensuring the privacy of survey respondents.

The questionnaire was designed in English then translated to the local spoken language Arabic by proficient speakers of both languages and was revised to be suitable to the general population.

The questionnaire used in the current study was developed to evaluate the participant's Knowledge, Attitude and Practice (KAP) regarding antibiotic usage and the related AR. In order to validate the questionnaire, a pilot run was performed using a group of 20 randomly selected individuals from each of the surveyed locations. The final version of the questionnaire used in this study was based on the results of the face validity and results of the pilot run.

### Study population (inclusion/exclusion)

Inclusion: We included males and females ≥18 years of age. Exclusion: People who were less than 18 years old, those who did not consent to participate, and those who live out of the region were excluded from the study.

### Questionnaire

The questionnaire contained nine questions divided into four sections. The first section collected demographic information of the participants. The second section contained two close-ended questions about accessibility to antibiotic therapy. The third section collected information about the practices of the participants with the antibiotic including: presence of an infection, duration of the use and methods of disposal of excess antibiotic. The last section collected information about AR awareness and their readiness to learn more about AR.

### Data storage

All data collection forms were kept in a secure setting, only available to the principal investigator, and will be destroyed by the principal investigator after completion of the study, in accordance with the requirement of the SREC at the University of Hail.

### Statistical analysis

All data were analyzed using MS Excel 2016 by means of descriptive statistics Chi-square analysis was used to calculate the p-value. Results with p-values less than 0.05 (p < 0.05) were considered statistically significant.

## Results

### Characteristics of respondents

All 500 participants (Table 1) completed the questionnaire. There was 285 female respondents (57%) with 312 (62%) of the respondents reported to have a college education.

**Table 1.** Demographic data.

Characteristic	Total respondents
Gender:	
– Female	285 (57%)
– Male	215 (43%)
Education:	
– Primary	9 (2%)
– Secondary	30 (6%)
– High School	112 (22%)
– College	312 (62%)
– Professional	37 (7%)

### Antibiotic access among participants

**Figure 1.**
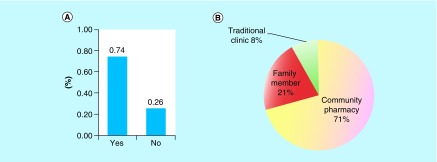
Prescription of antibiotics in Hail. **(A)** Percent of the participants who used an antibiotic that was prescribed (Yes) or not prescribed (No). **(B)** Sources of obtaining a non-prescribed antibiotic.

We relied on the participants to honestly self-report their access to antibiotics. We obtained 500 responses, some participants reported access to both prescribed and nonprescribed antibiotics. The latter refers to the practice when an antibiotic is accessed/dispensed by pharmacists in absence of a medical prescription. This is commonly practiced in many developing countries including Saudi Arabia as part of pharmacy malpractice that we described in an earlier study [13]. Nevertheless, out of the 500 responses, 370 (74%) of participants obtained their antibiotic with a prescription while 130 participants (26%) obtained their antibiotic without a prescription (Figure 1A). The participants who obtained their antibiotic without a prescription did so majorly from community pharmacies (71%), while others obtained their antibiotic from a family member (21%) or a traditional clinic (8%) (Figure 1B).

### Antibiotic use

**Figure 2.**
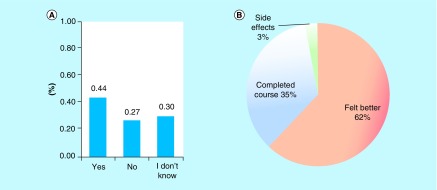
Antibiotic misuse in Hail. **(A)** Percent of the participants that consumed an antibiotic for a known infection (Yes), without a known infection (No) and unknown status of infection (I don't know). **(B)** Practices of antibiotic withdrawal.

To further understand the relevance of obtaining an antibiotic in the region of Hail, we asked the same participants to honestly self-report whether their use of the antibiotic was for a known infection. Among the 500 participants, 218 (44%) participants reported that they used the antibiotic for a known infection, while 133 (27%) participants used the antibiotic without a known infection and 149 (30%) participants were not sure if they used the antibiotic for an infection. We categorized the participants who were not sure of their infection status and who used the antibiotic without a known infection as one category and compared them to the respondents who took the antibiotic for a known infection. This resulted in a total of 282 (57%) participants who used an antibiotic without a known infection, compared with 218 (44%) participants who used an antibiotic for a known infection (p = 0.004; Figure 2A). To assess the general practices that come with antibiotic use among the participants in the region of Hail, we asked the participants to self-report on whether they completed the antibiotic course prescribed or counseled to them. Surprisingly, only 35% of participants (N = 173) completed the full course of the antibiotic (Figure 2B).

The majority of the participants stopped the antibiotic once they felt better. We also inquired about the fate of the antibiotic among the participants who did not complete the course or who were given a larger quantity than needed. Approximately 48% of participants threw away the remaining antibiotic while 42% stored it for future use (Table 2).

**Table 2.** Specification of the practices of the participants with left over antibiotics.

Criteria	Responses
Stored it	227 (45%)
Threw it	242 (48%)
Flushed it	11 (2.2%)
Gave it to a sick family member	18 (3.6%)
Returned it to pharmacy	4 (0.7%)
Other	2 (3.9%)

### AR awareness in Hail

**Figure 3.**
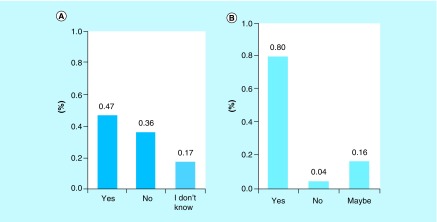
Awareness of antibiotic resistance and willingness to learn among study participants. **(A)** Participants knowledge of AR (Yes), no knowledge of AR (No) or unsure of AR (I don't know). **(B)** Participants willingness to learn about AR (Yes), not willing (No) and possibly willing (Maybe). AR: Antibiotic resistance.

We assessed the knowledge of the study participants regarding AR and whether they were aware of its rise. Among the 500 participants, 233 (46.6%) participants were aware of AR, 86 (17.2%) participants were not sure of AR and finally 181 (36.2%) did not know about AR and its rise (Figure 3A). The surveyed population was willing to become educated on AR and its rise, with a significant number of participants (80%) willing to learn more about AR (Figure 3B). A correlation analysis was conducted between the uses of antibiotics (infection status) versus knowledge about AR. A weak positive correlation was found between the two categories (data not shown).

## Discussion

There is a rapid increase in the emergence of antibiotic-resistant bacteria that threatens the efficacy of antibiotics, and puts the lives of millions of people at risk. While there is a growing interest in alternative drug sources such as those of plant origin or drug repurposing [13,14], there is a lack of interest in finding/introducing new antibiotics making AR a serious and concerning threat. AR was noted as early as the 1940s, where the minimum inhibitory concentration (MIC) for penicillin increased 10- to 50-times after being clinically used for 20 years [15]. AR is a multifactorial problem that includes the necessity of increasing public awareness about AR both locally and globally. Previous reports have documented the decreased efficacy of cephalsporins and the wide spread of resistance to expanded-spectrum cephalosporins in Saudi Arabia compared with 1988 [16]. Additionally, a recent national surveillance study evaluating the AR among various bacterial strains across hospitals in Saudi Arabia reported a significant number of antibiotic-resistant isolates [17]. The overuse or misuse of antibiotics can potentially contribute to the rapid development of AR, which is a global threat. As a result, the WHO devised a plan in 2015 that consists of five pillars, one of which is to improve awareness of AR, and Saudi Arabia was one of the countries that signed up to implement this plan [16].

Supporting other findings that demonstrated lack of appropriate antibiotic use in Saudi Arabia, the survey respondents of the current study, demonstrating a given population in Hail, obtained their antibiotics without a prescription (∼26%) majorly from community pharmacies [16,17]. The dispensing without a prescription by community pharmacies was also reported in the capital city of Saudi Arabia – Riyadh [16]. Community pharmacies dispense antibiotics without a prescription in many developing countries and programs that can reduce such activity or a policy that can be enforced should be taken seriously to reduce the access to ‘self-medication.’ Such a program has been shown to be successful in reducing the dispensing of antibiotics without a prescription in Spain [18].

According to the data obtained from our study, approximately 57% of respondents took an antibiotic without a known infection. This can lead to the unnecessary exposure of the bacteria to the drug, which can further increase the chances of acquiring resistance by gene mutation or horizontal gene transfer [15]. Restricting antibiotic use to only validated infections is not only important for Hail, but also for the global community [15,16]. Additionally, a great number of respondents reported withdrawing from an antibiotic course as soon as they felt better. Antibiotics are prescribed for a fixed amount of time, dose and dosage frequency [11]. The rationale behind an antibiotic course is the complete eradication of the pathogen; however, this has been argued and clinically assessed as to whether it is a beneficial approach to antibiotic use [3]. It is thought that extended exposure to antibiotics can contribute to AR itself [19]. However current practices recommend completing the full course and thus patients should be counselled to complete their full course of antibiotics. Interestingly, 42% of survey respondents reported to store antibiotics for future use. This practice can lead to unnecessary use of antibiotics, which can potentially increase AR.

Predictors of correct antibiotic use and knowledge of antibiotics have been found to be affected by education and source of information in Europe [20,21]. The majority of the participants of this study held a college degree (see Table 1), thus our population sample did not represent enough predicting factors to conduct such an analysis. However, the association between antibiotic use and the awareness of AR was weakly positive. The respondents that took the antibiotic for an unknown infection were more likely to be less aware of AR. Thus efforts focusing on educating the public about AR and correct antibiotic use are much needed in the region of Hail.

## Limitations & bias

The first drawback of the current study is that it was conducted in one region of Saudi Arabia, which does not necessarily reflect the general knowledge and practice of the country. Another potential limitation is that we did not investigate the type of antibiotics or the infections to which they are being used for. Also, we did not investigate, if any, side effects following the unregulated use of antibiotics, as these questions were beyond the scope of the study. Finally, the majority of our sample population held a college degree; this may have overestimated the actual practices of antibiotic use and knowledge about AR in the region of Hail.

## Conclusion

Our study highlights the necessity and need to increase public knowledge about antibiotic use and awareness about AR in the region of Hail. Additionally, enforcing community pharmacies to adhere to policies established to restrict antibiotic dispensing unless prescribed, encouraging antibiotic stewardship among healthcare practitioners, together with increasing public knowledge of antibiotic use will collectively contribute to the global efforts to reduce the threat of AR.

## Future perspective

AR represents a real worldwide challenge. Both physician prescription practice and patient knowledge are the main contributing factors that drive AR. Several studies, including the current one, have described a low patient knowledge about AR in Saudi Arabia. Therefore, unless there is a widespread community awareness about the issue, antibiotic misuse and AR will remain significantly high in the region. Also, there is a need for change in physician prescription practice in the region including the implementation of strict policies that limit antibiotic prescriptions. However, such measures will need many years to become an effective practice. Thus, we believe that there will not be major changes in practice and knowledge in the next decade or so.

**Summary points**

- Antibiotic misuse is widespread in the Hail region in the northern part of Saudi Arabia.
- Misuse of antibiotic leads to resistance, which represents a major threat to human health.
- Patient knowledge, attitude and practice are thought to contribute to antibiotic resistance.
- There is a low knowledge about antibiotic resistance in the region, but the majority are eager to learn.
